# Improving outcomes in chronic myeloid leukemia through harnessing the immunological landscape

**DOI:** 10.1038/s41375-021-01238-w

**Published:** 2021-04-08

**Authors:** Ya-Ching Hsieh, Kristina Kirschner, Mhairi Copland

**Affiliations:** Paul O’Gorman Leukaemia Research Centre, College of Medical, Veterinary and Life Sciences, Institute of Cancer Sciences, University of Glasgow, Glasgow, G12 0YN UK

**Keywords:** Myeloproliferative disease, Immunosurveillance

## Abstract

The quest for treatment-free remission (TFR) and deep molecular response (DMR) in chronic myeloid leukemia (CML) has been profoundly impacted by tyrosine kinase inhibitors (TKIs). Immunologic surveillance of residual leukemic cells is hypothesized to be one of the critical factors in successful TFR, with self-renewing leukemic stem cells implicated in relapse. Immunological characterization in CML may help to develop novel immunotherapies that specifically target residual leukemic cells upon TKI discontinuation to improve TFR rates. This review focuses on immune dysfunction in newly diagnosed CML patients, and the role that TKIs and other therapies have in restoring immune surveillance. Immune dysfunction and immunosurveillance in CML points towards several emerging areas in the key goals of DMR and TFR, including: (1) Aspects of innate immune system, in particular natural killer cells and the newly emerging target plasmacytoid dendritic cells. (2) The adaptive immune system, with promise shown in regard to leukemia-associated antigen vaccine-induced CD8 cytotoxic T-cells (CTL) responses, increased CTL expansion, and immune checkpoint inhibitors. (3) Immune suppressive myeloid-derived suppressor cells and T regulatory cells that are reduced in DMR and TFR. (4) Immunomodulator mesenchymal stromal cells that critically contribute to leukomogenesis through immunosuppressive properties and TKI- resistance. Therapeutic strategies that leverage existing immunological approaches include donor lymphocyte infusions, that continue to be used, often in combination with TKIs, in patients relapsing following allogeneic stem cell transplant. Furthermore, previous standards-of-care, including interferon-α, hold promise in attaining TFR in the post-TKI era. A deeper understanding of the immunological landscape in CML is therefore vital for both the development of novel and the repurposing of older therapies to improve TFR outcomes.

## Introduction

Chronic myeloid leukemia (CML) is a hematological cancer characterized by the presence of the BCR-ABL1 oncokinase resulting from the reciprocal translocation t(9;22) in myeloid stem cells of the bone marrow (BM). The current mainstays of treatment in CML are tyrosine kinase inhibitors (TKIs) of which imatinib, nilotinib, dasatinib, and bosutinib can be used as first-line treatments [1]. The development of TKIs has profoundly improved prognosis of chronic-phase CML patients, with treatment aiming to achieve a deep molecular response (DMR; MR^4^; *BCR-ABL1*mRNA ≤0.01%) to prevent disease progression. This enables TKI discontinuation in approximately half of patients who achieve DMR [2]. Quiescent and self-renewing leukemic stem cells (LSCs) have been implicated in refractoriness and disease progression. These LSCs can remain, even when there is no measurable residual disease, and upon changes in the BM microenvironment, can exit quiescence and drive relapse, representing a bottleneck to cure. Recently, it has also been suggested that immunologic surveillance of residual leukemic cells is a critical factor in maintaining treatment-free remission (TFR) [3–6]. It is thought that when treatment has removed the majority of CML cells below a certain threshold, the immune cells can limit the growth of residual leukemic cells leading to sustainable TFR. Therefore, immunological characterization and understanding of the mechanisms of immune cell interaction with CML cells in the BM may help to develop novel immunotherapies that specifically target residual leukemic cells upon TKI discontinuation to improve TFR rates. This review focuses on the immune dysfunction in newly diagnosed CML patients, the role of TKIs and other therapies in restoring immune surveillance, and the role of the immune system in maintaining TFR. Relevant terms used in this review are described in the Glossary (Table 1).

**Table 1:** Glossary of terms.

Term	Definition/Function
B-cells	Upon primary infection or immunization, a small population of antigen-specific B-cells becomes activated and expands after acquiring T-cell help. Some of these expanded clones then differentiate into memory B-cells.
CD8+ cytotoxic T-cells (CTLs)	T-cell exposure to antigens expressed on cancer cells and co-stimulatory signals leads to the development of effector function and T-cell clonal expansion. T-cell receptors on CD8+ T-cells bind to antigen, which is held in the major histocompatibility complex (MHC) complex on the surface of antigen-presenting cells, such as DCs. This then triggers initial activation of the T-cells.
Dendritic cells (DCs)	DCs efficiently process and present antigens to T-cells responsible for the initiation of immune responses.
Immune checkpoint: cytotoxic T lymphocyte antigen 4 (CTLA-4)	CTLA-4 is a cell-surface receptor, homologous to CD28, binding to ligands CD80/CD86 on antigen-presenting cells such as DCs. The binding of CTLA-4 to CD80 and CD86 is considerably stronger than the affinity of CD28, but unlike CD28 which activates T cells, CTLA4 delivers an inhibitory signal to T-cell activation. Thus, in cancer, CTLA-4 has undesirable effects that may prevent T cells from mounting a sufficient immune response.
Immune checkpoint: Programmed death 1 (PD1)	PD1 is one of the crucial immune checkpoint signals and is mainly expressed on mature CTLs. Interactions of PD1 with its ligand–PD ligand 1 (PDL1) reduce antigen-specific T-cell activation.
Leukemia-associated antigens (LAAs)	LAAs are immunogenic antigens which are able to induce specific T-cell responses and are target structures relevant for immunological targeting of leukemic cells.
Natural killer (NK) cells	NK cells lack T-cell markers but express CD56. Unlike T-cells, NK cells are not restricted to MHC-I/II molecules and can exert natural cytotoxicity against cancer cells based on signals from activating and inhibitory cell-surface receptors. CD56^dim^ cells are more differentiated and cytolytic. A subset of NK cells with CD57 expression further differentiates the functionally diverse CD56^dim^ subset and are considered ‘memory-like’ NK cells with higher cytotoxicity.
Natural killer cell receptors	NK cell receptors are classified into two types—‘inhibitory’ and ‘activating’. Major activating receptors involved in target leukemic cell killing are KIR2DS, natural cytotoxic receptors (NCRs)—NKp30, NKp46, and NKp80 and NKG2D. Tolerance of NK cells to normal cells is attained through their expression of MHC-I-binding inhibitory receptors including killer cell immunoglobulin-like receptor (KIR)2DL and natural killer group 2A (NKG2A).
Plasmacytoid dendritic cells (pDCs)	pDCs are a unique DC subset and produce large amounts of interferon (IFN)-α. Activated pDCs have strong antigen-presenting capacity, which plays an important role in NK cell recruitment and T-cell activation.

## An overview of immunological profile in CML and effects of TKI treatment

### Immune response to CML

CML is characterized by a period of immune dysfunction present in patients at diagnosis, prior to the commencement of TKI therapy. This facilitates tumor progression and self-preservation, by preventing host development of antileukemia immune responses. Innate immune responses including dendritic cells (DCs) [7], plasmacytoid dendritic cells (pDCs) [4], and natural killer (NK) cells [8], have been noted to be dysfunctional in CML patients, with reductions in cell count, cytotoxicity, and antigen-presenting function noted. Adaptive immune responses are dysfunctional in CML patients at diagnosis, including dysfunctional CD8+ cytotoxic T-cells (CTLs), and expressed/over-expressed leukemia-associated antigens (LAAs) such as proteinase-1 (PR1) and Wilm’s tumor-1 (WT1) [9–11]. In CML-bearing mice, CD8+ CTLs also displayed limited cytotoxic activity, and absence of interferon (IFN)-γ and tumor necrosis factor (TNF)-α production [12]. Additionally, CML CTLs had high expression of programmed death 1 (PD1), which interacts with PD1 ligand (PDL1) expressed on CML cells, leading to suppression of cell killing ability and subsequent disease progression [12]. Immune suppressor cells including myeloid-derived suppressor cells (MDSCs) and regulatory T-cells (Tregs) contribute to T-cell dysfunction and disease progression in CML, expanding at diagnosis, and reducing following TKI therapy [13–15]. Notably, MDSCs stemming from *BCR-ABL1* clones downregulate antitumor immune surveillance, by attenuating the action of NK and T-cells. These mediate their suppressive activity through increased reactive oxygen and nitrogen species, and depletion of arginine (through upregulation of arginase 1) and cysteine. The latter two amino acids being required for T-cell function and activation (by antigen-presenting cells such as DCs), respectively [16].

### Immune response after TKI treatment

TKIs have a dual mode of action with a direct inhibitory effect on BCR-ABL1 tyrosine kinase and immune-modulatory or suppressive effects. Contradictory results have been observed between in vitro and in vivo studies. Several in vitro studies have demonstrated inhibitory effects of imatinib and dasatinib on immune responses. Both imatinib and dasatinib reversibly inhibit T-cell proliferation in vitro but the effects of dasatinib are more profound [17, 18]. Furthermore, imatinib and dasatinib impair CD8+ CTLs specifically directed against LAA function in vitro [19, 20], and dasatinib also inhibits NK cell function [21]. In contrast to the in vitro results, clinical data showed that imatinib or dasatinib treated patients exhibit expansion of CD8+ CTLs or NK cells which are associated with an improved response to therapy [22–24]. Furthermore, dasatinib may induce a reversible state of aberrant immune reactivity, leading to large granular lymphocytic lymphocytosis, which is associated with a favorable clinical response [22]. These differences are likely due to the inability to recapitulate all aspects of the immune system and microenvironment in vitro.

### Role of immune cells in molecular response after TKIs

**Fig. 1:**
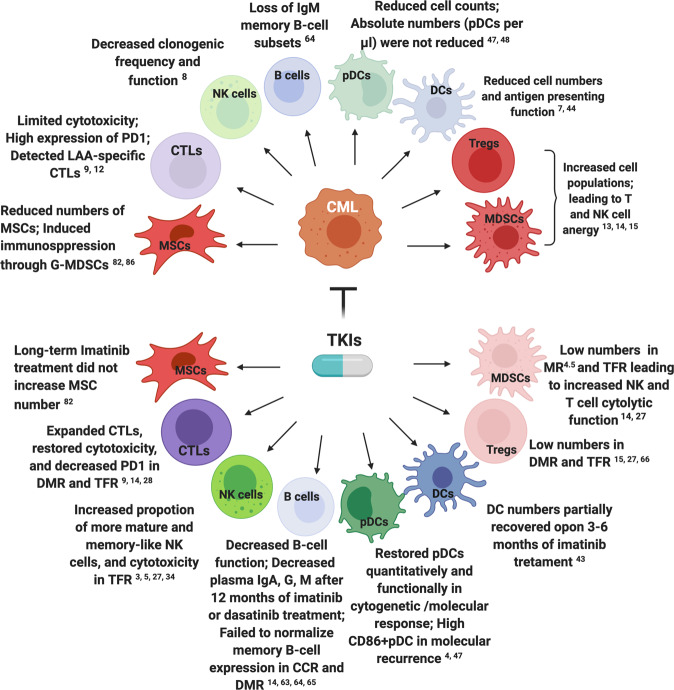
Immunomodulatory effects of tyrosine kinase inhibitors (TKIs) in chronic myeloid leukemia (CML) patients. Upper diagram—CML effects on untreated immune cells. Lower diagram—effects on immune cells after TKI treatment, including cytotoxic T-cells (CTLs), natural killer (NK) cells, dendritic cells (DCs) and plasmacytoid DCs (pDCs), myeloid-derived suppressor cells (MDSCs), regulatory T-cells (Tregs), mesenchymal stromal cells (MSCs) and B-cells. LAAs leukemia-associated antigens, G-MDSCs granulocyte-like MDSCs, PD1 programmed death 1, TFR treatment-free remission, DMR deep molecular response (or MR^4^; *BCR-ABL1* ≤ 0.01%), MR^4.5^ molecular response^4.5^ (*BCR-ABL1* ≤ 0.0032%), CCR complete cytogenetic remission. Illustration was created with BioRender.com.

Imatinib-treated patients in chronic-phase have ~20% chance of achieving DMR in the first 2–3 years of therapy, with the second generation TKIs dasatinib and nilotinib potentially permitting a more rapid DMR [25, 26]. The persistence of detectable leukemic cells while either on- or off-treatment in DMR are likely governed by immune-mediated control of residual disease. DMR is associated with increased NK and CD8+ T-cell numbers, and decreased MDSCs in the peripheral blood of CML patients [14]. Likewise, successful TFR has been linked to increased NK/CD8 T-cells, and decreased Tregs/MDSCs [3, 22, 27, 28], and low mature (CD86+) pDC frequencies [4]. In addition, the combination of IFN-α with imatinib has been demonstrated to improve outcomes [29, 30], with several clinical studies indicating that IFN-α in combination with TKI elicits a sustained DMR enabling possible TKI cessation [31–33]. The immunomodulatory effects of TKIs in CML patients are summarized in Fig. 1.

## Leveraging the immune system to enhance TFR: targeting specific immune functions

Enhancing or restoring immune effector functions may provide a pathway to maintaining and increasing TFR in CML. These approaches may be relatively nonspecific, e.g., IFN therapy, or may target specific immune functions. Here we discuss specific immune effectors including the innate system NKs, DCs, pDCs and their crosstalk, and the adaptive system such as CD8+ CTLs, LAAs, immune checkpoints, B-cells and immunosuppressive Tregs and MDSCs.

### Innate immune responses against CML cells

Innate immune cells can sense cancer cells, antigens, or changes in the microenvironment, leading to direct immune effector functions. NK cells and pDCs are the major innate immune cells and are considered crucial effectors of the antileukemic immune response. They act as the early warning system and a bridge to T-cell response or adaptive immunity.

#### Natural killer cells

A decrease in the clonogenic frequency and function of NK cells has been demonstrated in chronic-phase CML patients at the time of diagnosis, worsening with disease progression to accelerated- and blast-phase CML [8]. Furthermore, increased proportions of cytotoxic CD56^dim^ and memory-like CD57 NK cells are associated with successful TFR in CML patients after imatinib discontinuation [3, 5, 34].

#### Natural killer cell receptors

**Fig. 2:**
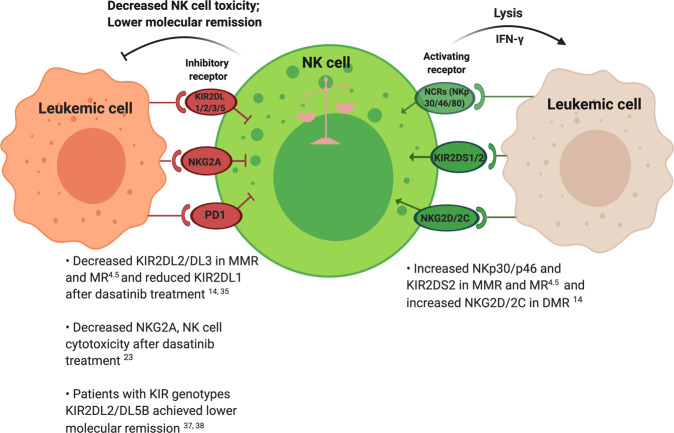
The major natural killer (NK) cell receptors affected in chronic myeloid leukemia (CML). Progression of NK cells from quiescence to activation is mediated by activating (in green) and inhibitory (in red) receptors. The balance between both receptor subtypes determines if NK cells are cytotoxic. The inhibitory killer cell immunoglobulin-like rectors (KIRs) and natural killer group 2A (NKG2A) receptor conduct inhibitory signals to restrain NK cell function to avoid killing normal cells under physiological conditions. In contrast, activating receptors such as natural cytotoxic receptors (NCRs)—NKp30, NKp46 and NKp80, and NKG2D trigger NK cell activation following binding to ligands upregulated on target cells undergoing stress and/or infection. PD1: programmed death 1. Illustration was created with BioRender.com.

The activation of NK cells is determined by the balance of signals delivered by activating and inhibitory receptors. NK cells are a heterogeneous population with respect to the expression of killer cell immunoglobulin-like receptors (KIRs), natural killer group 2 (NKG2), and natural cytotoxic receptors (NCRs) (Fig. 2).

#### Impact of TKIs on natural killer cell receptors

Treatment of CML patients with different TKIs alters the expression of various NK receptors. Dasatinib increases expression of inhibitory KIR2DL1 receptors, whereas imatinib increases expression of activating receptors including NKp30, NKp46, NKp80, and NKG2D [35]. Downregulation of inhibitory receptor NKG2A by dasatinib enhances NK cell cytotoxicity and results in more rapid treatment responses in CML patients [23]. NK receptors also play an important role in maintaining DMR and TFR, with expression of activating receptors NKp30, NKp46, NKG2A, NKG2C, and NKG2D restored to normal levels in molecular response compared with their downregulation at diagnosis [14]. KIRs have extensive polymorphisms, and it has been suggested that KIR genotypes correlate with NK cell immunity against CML and sustained DMR after discontinuing TKI treatment [6, 36]. Although no difference has been observed in KIR genotypes (such as KIR2DL2, KIR2DL5, and KIR3DS1) among non-relapsing, early and late relapsing groups in patients from the TFR clinical trial EURO-SKI [3], several studies have demonstrated that patients with genotypes KIR2DL5B and KIR2DL2 achieve lower rates of cytogenetic and molecular remission in response to TKIs, implying that specific KIR genotypes or high numbers of inhibitory KIR genes can suppress NK cell toxicity against CML cells [37, 38]. Furthermore, expression of KIR2DL2/DL3/DS2 was restored to normal levels following achievement of major molecular response (MMR; *BCR-ABL1* ≤ 0.1%) and molecular response^4.5^ (MR^4.5^, *BCR-ABL1* ≤ 0.0032%) compared with reduced levels at diagnosis [14].

A more recent study shows that acute myeloid leukemia (AML) LSCs that express stress-induced ligands NKG2DLs for receptor NKG2D are cleared by NK cells, whereas NKG2DL-negative LSCs isolated from the same individual escape cytotoxic NK cells. These NKG2DL-negative LSCs not only selectively survive chemotherapy but also evade immune surveillance [39]. Thus, evaluating both inhibitory and activating receptors to assess NK cell function overall would give a better indication of immune function in CML. Enhanced NK cell immunity, including NK cell receptors engaged with ligands expressed on CML cells, provides a promising immune therapeutic target to achieve TFR. NK cell modulating agents, such as lenalidomide, and the adoptive transfer of NK cells are currently undergoing clinical trials in other hematological malignancies. Lenalidomide shows potential in TFR by virtue of T and NK cell proliferation and activation, and enhanced NK cytotoxicity. A recent phase I trial showed a sustained *BCR‐ABL1* level ≤0.0032%, however, the trial was prematurely stopped due to concerns about thrombotic risk [40]. Interestingly, adoptive transfer of CNDO-109-activated allogeneic NK cells resulted in enhanced cytotoxicity and NK cell activation in high-risk patients with AML in phase I trials [41]. Alternatively, adoptively transferred cytokine-induced memory-like NK cells improved clinical responses in AML patients [42].

#### Dendritic cells

*BCR-ABL1*-positive DCs can be generated from peripheral blood mononuclear cells or CD34^+^ progenitor cells. They have an impaired capacity to capture and process antigens, and defective cell migration compared to DCs from healthy donors [7]. Research has shown that after 3–6 months of imatinib treatment, DC numbers partially recover but do not reach levels observed in healthy individuals [43]. However, there are contradictory findings regarding the capability of *BCR-ABL1*-expressing DCs in CML. In one study, DCs generated from blood progenitors of CML patients induced a primary CML-directed cytotoxic immune response in vitro [44]. In contrast, in another study, CML-derived DCs displayed reduced antigen-presenting function, low maturation status, and impaired homing to secondary lymphoid organs when compared to control DCs in a retroviral-induced murine CML model [45]. In AML, leukemic DCs also promoted T-cell anergy and the generation of Treg cells that are specialized in immune suppression, through upregulation of PDL1 on leukemic DCs [46]. Taken together, functional deficiencies of *BCR-ABL1*-expressing DCs may contribute to the escape from immunosurveillance observed in CML.

#### Plasmacytoid dendritic cells

pDCs have emerged as crucial effectors in innate immune responses in CML. Before treatment, CML patients have reduced numbers of circulating pDCs. Moreover, patients who were in complete cytogenetic or molecular response after imatinib treatment restored their blood pDCs both quantitatively and functionally and were comparable to healthy donors [47]. Contrary to previous studies, data from the EURO-SKI and German CML-V TIGER study recently showed that high numbers of activated CD86+ pDCs were observed in CML patients with molecular recurrence after TKI discontinuation. This was associated with T-cell exhaustion with increased PD1 expression on CD8+ CTLs and PR1-specific antileukemic CD8+ CTLs. The authors suggest that T-cell exhaustion with CD86+ pDCs contributes to recurrence risk and that low CD86+ pDC might be predictive of TFR [4]. Moreover, a recent concept was raised by Inselmann et al., based on the German CML-V study [48], in which low frequencies of *BCR-ABL1*+ pDCs (transdifferentiated from LSCs) may regulate antileukemic immunity in the early (pre-leukemic) evolution of CML as well as in DMR. However, high numbers of *BCR-ABL1*+ pDCs can also aberrantly express multiple inflammatory cytokines/chemokines that drive chronic inflammation, such as in CML patients with molecular recurrence, which stimulates CML-LSC persistence and immunosuppression. Using RNA sequencing, this study also revealed a strong inflammatory gene expression signature in CML-pDCs at diagnosis. Patients with high pDC counts (expanding with *BCR-ABL1*+ stem cell mass) at diagnosis achieved inferior rates of DMR under nilotinib, unless nilotinib therapy was combined with IFN, which strongly suppressed circulating pDC numbers [48]. The authors therefore suggest that high numbers of *BCR-ABL1*+ pDCs may be an early biomarker of failure to achieve DMR with reduced likelihood of TFR.

#### Interaction of natural killer and dendritic cells

NK-DC bi-directional crosstalk can lead to activation of both cell types including NK cell lysis of autologous DCs, production of proinflammatory cytokines, and stimulation of T-cell responses. A reciprocal relationship exists, with NK cells promoting DC maturation and activated NK cells having the ability to kill DCs that fail to undergo proper maturation through engagement of the activating receptor NKp30 [49]. In vitro studies have demonstrated that culture of activated NK cells with immature DCs at low NK/DC ratios promotes DC maturation, whereas a higher NK/DC ratio can result in NK cell-mediated killing of DCs [50]. This may add a new predictive indicator of immune system configuration for maintaining TFR. Moreover, it has been suggested that functionally defective pDCs interact with NK and T-cells leading to immunosuppression in the BM milieu in patients with multiple myeloma by engaging the PD1 signaling axis [51]. Future studies are needed to elucidate the interaction between NK cells and pDCs, and whether NK and T-cell functions are regulated by pDCs in CML after TKI cessation.

### Adaptive immune responses against CML cells

#### CD8+ cytotoxic T-cells

In CML at diagnosis, CD8+ CTLs are functionally exhausted with limited cytotoxicity, and impaired ability to expand and produce proinflammatory cytokines [52]. However, expansion of CD8+ CTLs was seen in CML patients treated with dasatinib, concomitant with an improved response to therapy [22, 53]. Furthermore, patients with DMR exhibited restored CD8+ CTLs with decreased inhibitory molecule PD1 [9, 14]. Patients who obtained TFR after IFN-α treatment also showed upregulation of CD8+ CTLs [54]. In addition, T-cell receptor-Vβ repertoire expression indicated T-cell dysfunction in CML patients, and an increase in the effector and memory CD8^+^ CTL fraction was reported in a CML patient maintaining DMR for 2.4 years after dasatinib cessation [55, 56].

CD62L, an effector memory T-cell marker, mediates T-cell trafficking to secondary lymphoid tissues and is correlated with treatment responses in CML. Its downregulation may impair effector CTL function in CML and abrogate antileukemic immune control. CD62L is shed from T-cells by the matrix metalloproteinase TNF-α-converting enzyme, and subsequently the soluble form of CD62L (sCD62L) can be detected in plasma. High CD62L+ expression on T-cells and concomitant reduced sCD62L levels were linked to superior molecular response to nilotinib therapy in CML. In the same study, TNF-α-converting enzyme was increased at diagnosis and significantly decreased during nilotinib treatment [57], therefore highlighting CD62L as a predictor of molecular response to TKI. Similar studies have shown that CD62L expression was effectively restored to normal levels in CML patients achieving MMR and MR^4.5^ on imatinib, nilotinib, and dasatinib [14]. Active immunotherapy, aiming at expansion and effector function of CTLs, may represent a powerful approach to target CML cells.

#### Leukemia-associated antigens

LAAs are over-expressed or aberrantly expressed in CML. CTLs target numerous LAAs on leukemic cells, including BCR-ABL1 fusion peptide, PR1, and WT1, which can lead to LAA-specific CTL induction, thus enhancing antileukemic immune responses. A blunted antigen-specific CTL response to LAAs was observed in CML patients at diagnosis. These immune effector responses were restored to normal levels in CML patients who achieved MMR and MR^4.5^ on TKIs and were retained in TFR patients who achieved a durable DMR on imatinib [14]. PR1 is an HLA-A2-restricted peptide derived from both proteinase 3 and neutrophil elastase. Induction of PR1-specific CD8 CTLs may contribute to sustained molecular response in CML patients on IFN-α maintenance after imatinib/IFN-α combination therapy and cytogenetic remission in CML patients after allogeneic hematopoietic stem cell transplantation (HSCT) [10, 31]. Moreover, among these LAA targets, WT1 is over-expressed in myeloid malignancies. A vaccine targeting WT1 was reported to be well-tolerated in AML patients with regression of measurable residual disease [58], and in an imatinib‐treated CML patient, WT1 vaccination also induced a reduction in measurable residual disease [11]. Furthermore, BCR-ABL1 peptide immunization induced an anti-BCR-ABL1 T-cell response that correlated with a subsequent decrease of at least 1 log of *BCR-ABL1* transcripts in imatinib-treated CML patients [59]. Lastly, CXorf48-specific CTLs, a novel LAA, were detected in patients who achieved TFR; in contrast CXorf48-specific CTL-negative patients had high molecular recurrence rates [60]. Vaccination with LAA-peptides may therefore induce specific expansion of CTLs, and together with safety demonstrated in clinical trials, in particular for WT1 [58, 61], highlights the importance of LAAs as relevant clinical immunotherapy targets in CML.

#### Immune checkpoint: programmed death 1

The three best described checkpoint molecules—PD1, T-cell immunoglobulin and mucin domain 3 (TIM3), and cytotoxic T lymphocyte antigen 4 (CTLA-4), act as inhibitory receptors of T-cells and have been associated with immune evasion in CML. CML-specific CTLs were characterized by the high expression of PD1, whereas their target leukemic cells expressed higher levels of its ligand, PDL1, in a CML mouse model and in patients [9, 12, 34]. PD1/PDL1 interactions contribute to functional T-cell impairment, which fails to eliminate measurable residual disease and may be related to leukemia relapse. Blocking this PD1/PDL1 interaction restored CML-specific CD8+ CTL function and prolonged survival of mice with blast-phase CML, suggesting that CD8 CTLs are crucially involved in the control of CML progression [12]. Furthermore, BM PD1 + TIM-3-CD8+ T-cells were correlated positively with failure to reach DMR [9]. Patients in MR^4.5^ also exhibited reduced PD1 expression on CD4+ and CD8+ T-cells, concomitant with increased NK and T-cell immune responses, suggesting that therapeutic methods to enhance NK cell function and immunogenic CTL responses or blocking aberrant PD1 signaling may result in greater success in TKI cessation studies [14]. Clinical trials to explore this in patients include the PD1 inhibitor nivolumab in combination with dasatinib (ClinicalTrials.gov Identifier: NCT02011945), and the PDL1 inhibitor avelumab in combination with TKIs in the ACTIW trial (ClinicalTrials.gov Identifier: NCT02767063). Although the mechanism of action in CML is uncertain, PD1 has recently been found to be expressed on NK cells. PD1/PDL1 blockade may therefore mediate some of its therapeutic effects through eliciting a strong NK response as demonstrated in murine cancer models [62].

#### Immune checkpoint: cytotoxic T lymphocyte antigen 4

In CML an increase in CTLA-4 expression on T-cells has been noted at diagnosis [9]. The EURO-SKI trial also recently reported that the CD86 receptor, ligand of CTLA-4, was increased on pDCs, and this was associated with exhaustion of CD8+ CTLs and higher recurrence risk after TKI cessation. This study raised the concept that PD1- or CTLA-4-blocking antibodies given directly prior to and temporarily after TKI discontinuation may block the immune inhibitory effects of pDCs on T-cells [4]. It is therefore plausible that CML cells may escape T-cell killing by promoting inhibitory feedback of CTLA-4 signaling, mediated via pDCs. A clinical trial (NCT01822509) is currently evaluating the efficacy of ipilimumab (anti-CTLA-4) in combination with nivolumab (anti-PD1) in patients with hematological malignancies, including CML.

#### B-cells

In CML, reduced immunoglobulin (Ig) levels and hypogammaglobulinemia were found in patients previously exposed to IFN-α and then treated with imatinib. The reduction of Ig levels was greater in patients with cytogenetic response, suggesting an effect of imatinib on dysregulation of B-cell function [63]. Moreover, loss of IgM memory B-cell subsets in CML patients at diagnosis and following complete cytogenetic remission on imatinib has been observed, suggesting that TKIs might interfere with the production and maintenance of B-cell memory [64]. Memory B-cell expression also failed to normalize despite achievement of DMR on TKIs [14]. Variable effects on Ig levels with TKIs include reduction in plasma IgA and IgG with imatinib, whilst IgM is reduced with dasatinib 12 months after treatment [65]. The impact of TKIs on B-cell response to influenza and pneumococcal vaccination in CML patients has found dose-dependent suppression of B-cell receptor signaling tyrosine kinases, such as Bruton’s tyrosine kinase, and downstream phospholipase C-γ-2. These kinases are essential in B-cell signaling and survival, resulting in a TKI-induced, off-target kinase impairment of B-cell responses in CML patients [64].

### Immunosuppressive cells—T regulatory and myeloid-derived suppressor cells

Tregs and MDSCs represent two immunosuppressive cell populations increased in CML patients at diagnosis; they elicit T and NK cell anergy, inhibiting immune-mediated attack against leukemic cells. Imatinib, dasatinib, and nilotinib attenuated the number of Tregs and MDSCs in patients with CML. Moreover, low numbers of Tregs were observed in patients achieving DMR following dasatinib treatment, with Tregs showing a strong inverse correlation with NK cell differentiation, indicating that inhibition by dasatinib enhances NK cell-mediated killing of leukemic cells [15]. Reduced Tregs were also associated with successful maintenance of TFR in CML [66]. Furthermore, reduced numbers of monocytic MDSCs were associated with increased NK and effector T-cell cytolytic function, and decreased T-cell PD-1 expression in CML patients with MR^4.5^ following TKI treatment [14]. It is known that MDSCs can suppress effector T-cells as well as induce Tregs via the aberrantly expressed PD1/PDL1 pathway, thus promoting tumor development. MDSCs were found to be part of the tumor clone displaying *BCR-ABL1* expression [67]. It is likely that TKIs target MDSCs that are part of the *BCR-ABL1*-positive leukemic clone and inhibit Treg activation, thus restoring NK cell killing and effector T-cell function via PD1 signaling [14]. Beyond the direct suppressive effects of MDSCs on NK and T-cells, other indirect mechanisms have been identified, including accumulation of reactive oxygen species and nitric oxide, and depletion of L-arginine and cysteine [67].

## Leveraging the immune system to enhance TFR in CML: nonspecific immune approaches

Targeting of specific immune effectors to enhance or sustain TFR in CML is one of the goals of precision oncology. However, the repurposing of traditional, nonspecific modulators of the immune system such as IFN-α, donor lymphocyte infusion (DLI), and mesenchymal stromal cells (MSCs) in the BM microenvironment, are emerging as therapeutic targets in the context of TFR in CML.

### Activating effector immune cells by IFN-α therapy

Prior to imatinib, IFN-α was the gold standard therapy for CML patients, with a small proportion of patients undergoing treatment with IFN-α alone maintaining durable remission. Of note, a percentage of these patients may sustain long-term durable remissions after discontinuation of treatment [68]. In contrast to imatinib, evidence suggests that IFN-α preferentially targets LSCs responsible for CML recurrence. Angstreich et al. showed that IFN-α was toxic to the primitive progenitors from CML patients responsible for the maintenance of long-term cultures, whereas imatinib preferentially targeted more mature and differentiated CML progenitors that constitute the bulk of leukemia [69]. These findings may underlie the slower but more persistent clinical effects of IFN-α in comparison to imatinib. Data from a mouse model also showed that IFN-α activates dormant hematopoietic stem cells (HSC)—exiting G0 phase and entering an active cell cycle, and thus sensitizing them to subsequent killing by 5-fluoro-uracil [70]. In addition, IFN-α may attenuate CML by activating PR1-specific CD8+ CTLs and NK cells, and inducing DC differentiation with specific antileukemic function [10, 31, 71]. Therefore, IFN-α has made a comeback as a viable therapeutic option in CML for its unique activity against dormant CML stem cells and activating the specific immunity that is essential for maintaining DMR and possible TKI cessation. Recently, several clinical trials have found that the use of IFN-α in combination with TKIs elicits a sustained DMR and potential for TKI discontinuation [31–33]. Interestingly, CML patients receiving upfront IFN-α plus imatinib therapy, followed by low-dose IFN-α maintenance, showed low rates of early progression and a trend towards deepening of their molecular response [31]. A median 8-year follow-up of the same patient cohort reported that IFN-α enabled therapy discontinuation in most patients provided they had been in MMR at the time of imatinib discontinuation [33]. However, more recently a study reported that CML patients with long-term combination treatment of IFN-α and TKI (median 3.4 years) exhibited an enhanced immunosuppressive state which was associated with upregulation of immunosuppressive cells including Tregs, MDSCs and CD4+ PD1+ cells, and expansion of immature NK cells [72]. Co-administration of IFN-α may therefore offer a strategy to support long-term TKI discontinuation, but must be counterbalanced by close monitoring of immunosuppressive cell subsets.

### Leverage existing immunological approaches—donor lymphocyte infusion

DLI is the treatment of choice for CML patients in relapse after allogeneic HSCT, with the seminal work of Kolb et al. demonstrating the profound efficacy in inducing remission and cure in chronic-phase CML [73]. Allogeneic HSCT remains an important rescue strategy for CML patients who do not respond adequately to TKI therapy or patients with advanced phase disease. DLI has strong antileukemic effects, and can induce a direct graft-versus-leukemia (GVL) reaction and restore complete remission for CML patients who relapse after allogeneic HSCT [74]. Graft-versus-host disease (GVHD) and marrow aplasia are the two major complications of DLI, which are greatly reduced with escalating dose regimens, while preserving the GVL effect. Responses to DLI, including the incidence of GVL and GVHD in CML, have been discussed elsewhere [75].

In recent years, TKIs have been used as pretransplant and maintenance therapy following allogeneic HSCT in CML, especially in advanced phase disease. Historically, in TKI naïve patients, imatinib resulted in higher rates of overall and disease-free survival than DLI in CML relapse after HSCT [76]. TKIs were also found to be better tolerated than DLI and produced durable remission in most patients with CML who have relapsed disease, especially chronic-phase relapses, after HSCT [77]. However, the ability to prescribe a TKI post-HSCT is dependent on whether or not a patient has demonstrated resistance/intolerance to all available TKIs pre-transplant, and this may limit therapeutic options, likely making DLI preferable. In terms of synergistic effects, contradictory results have been published. More than a decade ago, several small studies demonstrated benefit in combining DLI with TKI in CML relapse after HSCT such that rapid and durable remissions were observed [78]. However, more recent studies have demonstrated that TKI-only had the highest cumulative incidence of complete molecular remission and lowest cumulative incidence of death when compared to DLI alone or TKI + DLI, although these results failed to reach statistical significance, suggesting that TKI salvage continues to provide significant survival in CML relapse following HSCT [79, 80]. Taken together, the data on the comparison between TKI and DLI are still limited and the exact role of TKI in CML relapse post-HSCT needs to be further defined.

### Immunomodulator mesenchymal stromal cells

MSCs, mainly from BM, have immunoregulatory and immunosuppressive roles in promotion of tumor growth and drug-resistance in CML. They are considered critical contributors to leukemogenesis and the protection of CML-LSCs from TKI therapy. Although MSCs from CML patients do not express BCR-ABL1, they may play an essential role in the activation of alternative survival signaling pathways, such as the Wnt pathway, indirectly protecting leukemic cells from therapy [81]. MSCs from untreated CML patients, or from patients on long-term treatment with imatinib, had a lower frequency but otherwise normal functional integrity. In vitro studies indicate that these suppressive effects of imatinib on MSCs are reversible, with MSC proliferation recovering on drug discontinuation [82]. This has implications for both the bone remodeling side effects and duration of treatment with imatinib. Gene expression profiling of MSCs from CML patients at both diagnosis and achievement of DMR after imatinib or dasatinib treatment, found similar gene expression aberrations at both stages. This study suggested that MSCs exhibited an abnormal gene expression pattern which might have been established during leukemogenesis within the BM niche and persisted in patients with DMR [83]. Recently an in vitro study reported that MSCs promoted TKI-resistance through a BCR-ABL1-independent mechanism via upregulation of the IL-7/JAK1/STAT5 pathway, with IL-7 or JAK1 inhibition sensitizing CML cells to TKI therapy and reducing resistance [84].

MSCs also play a pivotal role in maintaining normal HSCs and LSCs within the BM niche. CXCL12 is the major chemoattractant for maintenance of HSCs and development of B-cells, pDCs, and NK cells, and plays a major role in their localization to regulatory niches. In CML patients, CXCL12 levels are reduced in the BM niche and only partially restored after imatinib treatment. Furthermore, targeted deletion of CXCL12 from MSCs reduced normal HSC numbers but promoted LSC expansion by increasing LSC self-renewing cell divisions, possibly through enhanced EZH2 epigenetic activity. The authors concluded that in CML, the CXCL12-expressing MSC niche maintains LSCs in a quiescent, TKI-resistant state [85]. Furthermore, MSCs from CML patients induced immunosuppression by reducing T-cell proliferation via granulocyte-like-MDSCs. It was suggested that MSCs over-expressing immunomodulatory factors—TGFβ, IL-6, and IL-10, could be involved in MDSC activation [86]. This supports the concept that MSCs contribute to CML disease development and progression through immunosuppressive properties. Additionally, MSCs in AML have been observed to deliver functional mitochondria to leukemic cells, in particular during chemotherapy, thus enhancing leukemic cell survival rate. Cytochalasin blocks this transfer, supporting the potential involvement of nanotubes in this process [87]. These findings are further supported by evidence that LSCs from TKI-resistant CML patients also rely on mitochondrial transfer. Together these data have led to the clinical use of tigecycline in combination with imatinib for TKI-resistant CML [88].

## Immunotherapy in TKI-resistant mutations and blast-phase patients

### IFN-α

As monotherapy or combination with TKIs may provide scope to mitigate TKI-resistant mutations and maintain response in CML. A TKI-resistant patient treated initially with IFN-α monotherapy for 8 months lost the T315I mutation but acquired a new F359V mutation. Further combination treatment with dasatinib resulted in complete hematologic response [89]. Another patient with hematological relapse due to T315I mutation after prolonged (35 months) imatinib treatment, underwent subsequent IFN-α monotherapy for 10 weeks, resulting in sustained DMR [90]. IFN-α individualized therapy in four patients with dasatinib-resistant T315I or M351T/F317L mutations, led to MMR or DMR whilst reducing mutations to undetectable levels. The authors suggested that the principal mechanism underlying this success was biphasic—with immune activation induced by dasatinib pre-treatment followed by restoration of immunological surveillance after application of IFN-α therapy [91]. In vitro, IFN-α combined with arsenic trioxide, synergized to inhibit proliferation and induce apoptosis of imatinib-resistant CML cell lines, whilst in vivo murine models showed significantly prolonged survival of primary T315I-CML mice whilst dramatically impairing disease engraftment in secondary mice [92]. IFN-α may thus provide an alternative therapeutic approach in selected TKI-resistant mutation positive CML patients, particularly those ineligible for alternative options such as BM transplantation.

### Chimeric antigen receptor T-cells

Chimeric antigen receptor (CAR)-T-cells are one of the emerging clinical success stories in B-cell malignancies, and translating this success to the field of myeloid malignancies holds promise. IL1 receptor accessory protein (IL1RAP), a co‐receptor for the IL1 and IL33 receptors, is an intriguing rare cell-surface marker that is expressed on CML cells but not normal HSCs [93]. Both in vitro and in vivo studies suggest that CAR-T-cells targeting IL1RAP specifically kill quiescent CML stem cells [94]. Additionally, anti-IL1RAP-CAR-T therapy has a favorable side effect profile, with no major deleterious effects on healthy hematopoietic cells both in vitro and in vivo, and without some of the adverse events more commonly associated with CAR-T therapy such as off-target toxicity or tumor lysis syndrome. A combined CAR-T and TKI approach has also been used in limited clinical cases to eliminate CML stem cells. A lymphoid blast-phase CML patient harboring T315I mutation achieved complete molecular remission and returned to chronic-phase with combination anti-CD19 CAR-T therapy and dasatinib treatment. The authors hypothesize that anti-CD19 CAR-T cleared the T315I mutation through elimination of all CD19+ cell clones, which resulted in clinical re-sensitization to dasatinib [95]. Anti-CD19-CAR-T-cells may also exhibit potent cytotoxic activity against RUNX1 mutations in blast-phase CML patient cells, with an additive effect to imatinib. This has been observed ex vivo in both a *RUNX1*^*pR204Q*^ mutated patient who carried T315I resistance mutation, and in a *RUNX1*^*pR162K*^ mutation with no TKI-resistance mutation [96]. In the quest to eradicate CML LSC, CAR-T-cell therapy represents a paradigm shift in conventional anti-cancer chemotherapy. CAR-T-cells may confer particular therapeutic benefit to TKI-resistant/intolerant, young or advanced phase CML patients.

### Other targeted therapies

Other targeted immunotherapy approaches are currently in development, including the anti-CD33 antibody gemtuzumab ozogamicin in myeloid blast-phase, and blinatumomab, a bispecific anti-CD3/CD19 T-cell engager (BiTE), consisting of bivalent bispecific antibody variable fragments linked together [97]. This creates a molecule with distinct binding properties at each end, with one binding CD3 on the surface of T-cells, and the other CD19 on B-cells. Blinatumomab is approved for use in relapsed B-cell acute lymphoblastic leukemia (B-ALL) and may also have a role in treatment of lymphoid blast-phase CML.

## Differences and similarities of immune profiles in CML versus other leukemias

Comparing immune profiles in CML and other leukemias may provide insight into future shared treatment strategies. Furthermore, the majority of studies on CML immunology have been conducted with peripheral blood samples, with BM proposed as the standard tissue for immuno-oncological studies as it represents a particularly immunosuppressive microenvironment in CML [9]. One such area investigated recently is the BM immunological landscape of CML, AML and B-ALL patients at diagnosis compared to heathy donors. Using multiplex immunohistochemistry and computerized image analysis [98], Bruck et al., found that immune checkpoint lymphocyte-activation gene 3 + T-cells were depleted in CML, AML and B-ALL. However, in CML the expression pattern of immune checkpoint TIM3 differed from other immune checkpoints by being notably enriched compared with other leukemias (AML, B-ALL) or healthy donors. The reason for this contrast remains unknown but might provide the basis for novel immunomodulatory approaches or immune checkpoint inhibitors. Additionally, this study also observed a higher macrophage polarization toward M1 and M2 phenotypes and an abundance of MDSC-like cells in CML BM, compared with AML or healthy BM, although the authors recognized that the MDSC-like cells may partly represent expanded malignant CML cells. Previous studies by Bruck et al., demonstrated that TKI treatment responses are more potent in patients with less exhausted T-cells. Although this may simply reflect disease aggressiveness, the authors’ note that this may also be due to off-target immunomodulatory effects of TKIs enhancing pre-existing antileukemic immune responses or dampening immunosuppressive cells such as Tregs. Perhaps more importantly, BM T-cells may provide a means of predictive modeling in CML, with regression modeling indicating that low CD4+ T-cell proportion, high PD1 + TIM3-CD8+ T-cell proportion, and high neutrophil count in peripheral blood are predictors of poorer TKI response [9]. Furthermore, comparative immune profiling in leukemias points towards the development of immunohistochemical scoring tools for predicting TKI response in CML.

## Nonimmune mechanisms of TFR loss in CML

Although this review focuses on immune mechanisms of TFR failure, nonimmune mechanisms of TFR escape including residual LSCs, quiescent LSCs insensitive to TKI, and senescence/clonogenic exhaustion of BCR-ABL1 cells, are nonetheless closely intertwined with immune function. LSC persistence, both on-therapy and off-therapy during TFR, have been noted in the majority of CML patients [99]. In TFR specifically, the majority of failures occur within the first 6–12 months, implicating residual LSC populations in these early recurrences, suggesting control but not elimination by TKI therapy. Recently, results of long-term follow-up of patients from the Imatinib Suspension and Validation study, a multi-center trial of imatinib discontinuation in CML patients with undetectable DMR, found an inverse relationship between patient age and risk of relapse, with the suggestion of age-related exhaustion of clonogenic capacity of BCR-ABL1 quiescent CML cells. Additionally, the majority of non-relapsed cases had at least 1 positive BCR-ABL1 transcript, indicating an unknown mechanism for self-limiting growth of leukemic cells despite treatment absence [99]. TFR loss may also be viewed as a stochastic event, with processes underlying HSC division unknown. Therefore, the quiescent BCR-ABL-positive LSC only becomes of clinical significance when the cell divides, with resulting loss of TFR. Some data suggests that this occurs by chance, and the loss of TFR after TKI discontinuation has been likened to the concept of cancer latency, although the two processes are biologically distinct [100].

## Conclusions

The emerging landscape of immune dysfunction and immunosurveillance in CML highlights the critical importance of the immune system in optimizing treatment responses in CML. Newer precision oncology approaches targeting specific immune functions, such as LAA vaccines, immune checkpoint PD1 inhibitors, and CAR-T-cell therapy hold great promise. Intriguingly, previous standard-of-care therapy, IFN-α, exhibits nonspecific, untargeted effects on the immune system, has been repurposed to enhance TFR in the TKI era. Together, specific and nonspecific immune effectors may make the concept of “operational cure” a reality for the vast majority of patients in the coming decade.
